# The Etiology of Empyema

**Published:** 1890-12-13

**Authors:** 


### THE ETIOLOGY OF EMPYEMA.

The belief that ordinary serous pleurisy could pass into the purulent form, or that empyema might be excited by chills in the same way as catarrhal pleuritic inflammation, has been generally abandoned by scientific physicians; but the bacterial origin of all empyemas, whether tubercular or not, was first promulgated by that distinguished clinicist, Professor Bacelli, of Rome, and has naturally attracted the special attention of the Italian physicians. Dr. Netter, however, has recently contributed to the *Revue Gén. de Clin. et de Thérap.* a report of the results of the examination of the effusion in 109 cases of purulent pleurisy. In twelve the B. tuberculosis was present, in fifty-one streptococci, in thirty-two pneumo-cocci, and in fourteen only were two or more forms of microbes found associated. The overwhelming proportion in which one species only existed points irresistibly to the direct relation between it and the particular form of the disease, the co-existence of those of tuberculosis and another being easily explained by the occurrence of the latter form in an individual already the subject of tubercular disease of the lungs. Clinical observation lead him to the conclusion that the empyema due to pneumo-cocci, which is most frequent in children, is the least grave, often recovering by spontaneous evacuation of the pus. In such cases he recommends its removal by repeated aspiration, incision being resorted to only when aspirations fail. Streptococcal pleurisy he found most frequent in adults and of far graver character. In these aspiration rarely sufficed, and incision, with the most complete evacuation of the purulent effusion, was almost invariably necessary. He lays great stress on repeated washing out of the pleural cavity with powerful bactericides, as sublimate, on account of the rapidity with which the streptococci, that may have escaped previous injections, multiply and maintain the infective process. Putrid pleurisies call for similar treatment. Tubercular pleurisies do not heal by incisions, but are relieved by aspirations performed at long intervals. These observations demonstrate the duty incumbent on every medical man to practise the bacterial examination of the effusion obtained by exploratory aspirations in each case, since in the one class aspiration is all that is required, in the second complete evacuation by incision and thorough antisepsis are imperative, while in the third, or tubercular, such an operation can do no good, and must accelerate the fatal termination.

